# The Nottwil Standard-Development and Implementation of an International Classification of Functioning, Disability and Health-Based Clinical Standard Assessment for Post-acute Rehabilitation After Newly Acquired Spinal Cord Injury

**DOI:** 10.3389/fresc.2021.720395

**Published:** 2021-09-13

**Authors:** Anke Scheel-Sailer, Patricia Lampart, Melissa Selb, Michael Baumberger, Hans Peter Gmünder, Diana Sigrist-Nix, Klaus Schmitt, Gerold Stucki

**Affiliations:** ^1^Swiss Paraplegic Centre, Nottwil, Switzerland; ^2^Department of Health Sciences and Medicine, University of Lucerne, Lucerne, Switzerland; ^3^Swiss Paraplegic Research, Nottwil, Switzerland; ^4^ICF Research Branch, Nottwil, Switzerland

**Keywords:** rehabilitation, post-acute care, spinal cord injury, international classification of functioning, disability and health, functioning, assessment 2

## Abstract

**Introduction:** Assessments during rehabilitation of spinal cord injury (SCI) align with the World Health Organization's classifications and national quality requirements. This paper aims to report on the development and first implementation experiences of an institutional standard of assessments performed after newly acquired SCI.

**Setting:** Specialized SCI acute care and post-acute rehabilitation clinic in Switzerland.

**Methods:** A situation analysis of an interdisciplinary post-acute SCI rehabilitation program was performed. The results informed a subsequent consensus-based selection of assessments, and an information and implementation strategy. Linking to the ICF Core Set for SCI in post-acute settings and ICF Generic-30 Set was performed. The Nottwil Standard was piloted for 18 months.

**Results:** Situation analysis: A battery of 41 assessments were irregularly performed during initial rehabilitation after newly aquired SCI. Selection of assessments: A multidisciplinary group of clinicians agreed on 10 examinations, 23 assessments and two questionnaires that make up the Nottwil Standard. In total, 55 ICF categories are covered, including most of the ICF Generic-30 Set categories. The implementation strategy included Executive Board commitment, a structured improvement project, guidelines for documentation and assessments, a manual controlling system, and staff training on the Nottwil Standard. Pilot phase: 54 persons with paraplegia and 42 with tetraplegia (75 male; 21 female) were included. Twenty-seven assessments out of 33 assessments were performed in more than 80% of all observed patients' rehabilitation.

**Conclusion:** Implementation of a standard assessment schedule was feasible but required a well-structured process with good communication strategy and controlling mechanism, and full engagement of involved professions.

## Introduction

Quality clinical care of persons with spinal cord injury (SCI) after a newly acquired SCI demands a comprehensive and accurate assessment of their medical and functioning needs (1–4). A major challenge is determining the assessment tools, clinical examinations and other sources of information (collectively referred to as “assessment tools” from now on) to employ in the assessment. Ideally, such a battery of assessment tools is part of standard assessment procedures and reflects current rehabilitation practice. The development of such a standard should rely on a number of principles that guide its development.

### Guiding Principles

The first guiding principle is the application of WHO classifications, specifically the International Classification of Diseases (ICD) (5) and the International Classification of Functioning, Disability and Health (ICF) (6), to meet the objective of SCI rehabilitation, i.e., optimization of the person's functioning (2, 7). The ICF is central to the development of an assessment standard that relies on a four-step approach of standardized documentation of functioning (8). While the ICD can be used to diagnose disease and injury, the ICF can be used to describe the functioning of a person from a bio-psycho-social perspective. The ICF encompasses categories that are hiearchically organized under the following components: body functions (e.g., shoulder pain, muscle tone functions in wheelchair-using patients with SCI), body structures (e.g., shoulder joint or arms), activities and participation (e.g., moving around using a wheelchair, work, participation in wheelchair sports) and environmental factors (e.g., barrier-free buildings). ICF categories contain a letter, i.e., b for body functions, s for body structures, d for activities and participation and e for environmental factors) and a series of numbers representing the four levels of detail in the hierarchy, from least detailed chapter level (e.g., b2 Sensory functions and pain) to the most detailed fourth level (e.g., b28014 Pain in upper limb) (6).

The four-step approach encompasses deciding on (1) what ICF domains (or categories) to document; (2) what perspective to take; (3) what data collection tools to apply; and 4) which approach to use for reporting. ICF Core Sets, short lists of ICF categories for specific health conditions and settings (9, 10), can be used to define what to document. The ICF Generic-7 and Generic-30 Sets as a minimum set of categories independent of health condition and setting, can also be used in the first step and across the care continuum (11, 12). For the rehabilitation care of persons with newly acquired SCI, the ICF Core Set for SCI in post-acute care (13) would be most appropriate to use. This ICF Core Set was developed in a multiple stage consensus process in which experienced SCI specialists from different professions and countries across the world defined the most relevant categories that can be used during this rehabilitation phase.

The ICF is also key to the second principle, i.e., aligning with international initiatives to scale up rehabilitation. A trailblazing initiative has been led by the Physical and Rehabilitation Medicine (PRM) Section and Board of the European Union of Medical Specialists (UEMS-PRM) that reflects the crucial interaction between practice, science and governance (policy) (14). The UEMS-PRM implementation action plan calls for, among other things, the “identification of data collection tools that cover the ICF domains included in the clinical assessment schedules [CLAS] of specific rehabilitation service [types]” (14–16). Aligned with the aforementioned four-steps approach, a CLAS is the specification of functioning aspects to document [using ICF Core Sets (9, 10) and ICF Generic Sets (11, 12)], for whom and when, and the data collection tools to employ (16, 17). Given that a CLAS designated for a specific health condition should cover all relevant aspects of functioning and contextual factors relevant for persons with that health condition, the ICF Core Set for SCI in post-acute care should be used for specifying the CLAS for the rehabilitation of persons with newly acquired SCI. Assessment tools should be administered as soon as possible at the beginning and at the end of an intervention (17). The UEMS-PRM action plan also calls for developing national ICF-based rehabilitation quality management strategies that are consistent with existing clinical quality management systems (14). This is related to the third principle.

The third principle is the consideration of national clinical quality management requirements for the reporting of health data. In Switzerland, functioning data from rehabilitation institutions are reported to the Swiss National Association for Quality Development in Hospitals and Clinics (ANQ) [https://www.anq.ch/de/fachbereiche/rehabilitation/]. In addition to functioning data, health condition(s), the Swiss Classification of Operations (CHOP) codes for interventions and procedures (18, 19), and the definition and achievement of rehabilitation goals according to ANQ criteria (20).

The fourth principle is the consideration of evidence provided by the SCI-specific cohort studies, e.g., Swiss SCI cohort study (SwiSCI; https://www.swisci.ch) and the European Spinal Cord Injury cohort study (EMSCI; https://www.emsci.org), and SCI-specific research platforms and resources on outcomes, e.g., the Spinal Cord Injury Research Evidence (https://scireproject.com/ and) or the Spinal Cord Outcomes Partnership Endeavor (SCOPE) (21, 22) in developing robust assessment standards. Especially relevant for developing standards for the assessment of persons with newly acquired SCI are the S2e guidelines for outcome measures in initial rehabilitation after the onset of a SCI (23, 24) led by the German-speaking Medical Society of Paraplegia (DMGP). Scientific evidence also encompasses information about validity and reliability of assessment tools (25).

The fifth principle relates to the implementation of the assessment standard. For an assessment standard to be feasibly implemented, it should adhere to the requirements of insurers, i.e., that the provision of care is suitable, feasible, cost-efficient and is based on scientific evidence (26). Essential for effective implementation of an assessment standard are health professionals trained in applying the tools contained in the assessment standard, including knowing the appropriate timing for applying specific assessment tools (27), as well as care provider leaders who support its implementation (28). Moreover, the feasibility of implementating an assessment standard is enhanced with the availability of a supportive infrastructure, e.g., health information system (HIS) and administrative support (28).

### Initiating the Project

The impetus for developing the Nottwil Standard is illustrative of what UEMS-PRM highlights as the interaction between practice, science and governance (policy). The clinic's decision-makers recognized the need to implement international recommendations for outcome measures and evidence-based rehabilitation of persons with newly acquired SCI (practice), and to meet requirements of financing-relevant stakeholders (e.g., insurances) for rehabilitation quality as well legal requirements (governance/policy). Furthermore, the clinic and its partner research institute envisioned the translation of cohort study results, namely from SwiSCI and EMSCI in rehabilitation quality management. The decision to initiate the project was made at a workshop of clinic and research institute leaders in 2015. Subsequently, a workshop was held in January 2016 at the research institute, during which the UEMS-PRM implementation action plan was developed (14).

The objective of this paper is to report on the development of an assessment standard (called Nottwil Standard) for use in the rehabilitation of persons after newly acquired SCI according to the guiding principles and to report on the first experiences in implementing it.

## Methods

### Design

This study is an implementation study starting with an observational situational analysis, presenting the participatory consensus process and ending with an observational analysis after the implementation of the newly developed standard.

### Setting

This project took place in an acute inpatient rehabilitation and outpatient clinic specialized for SCI and under the auspices of its integrated quality management and multiproject management unit. The clinic is part of a larger organization that also includes a research institute and is governed by a foundation. Since 2006 the clinic has been developing an ICF-oriented culture, striving to increasingly implement ICF elements in interprofessional clinical management.

### Preparatory Activities

#### Situation Analysis

An observational study, a situation analysis was conducted that involved an analysis of retrospective data from patients (adults ≥18 years old) admitted for rehabilitation after newly acquired SCI from December 2014 to December 2015 (3, 27). Among the data analyzed were the assessment tools used by physicians, physiotherapists, occupational therapists and nurses, and assessment procedures, including adherence to administration recommendations (e.g., timing). The situation analysis results were considered in the development of the Nottwil Standard.

#### Developing the Nottwil Standard

The project was led by a rehabilitation physician with experience in rehabilitation quality management research methodology and conducted by a multidisciplinary core project team (CPT) consisting of the project leader, the rehabilitation department head, the chief physician of rehabilitation, the head of corporate development and scientific assistant. The CPT was supported by an expanded project team (EPT) representing all relevant professions involved in the routine SCI rehabilitation process, including peer counselors, who represented the perspective of a person with SCI. The development of the Nottwil Standard was driven by an inclusive and consensus-based approach. Content and milestones were discussed, revised and approved by the CPT and EPT. The overall project proceeded in alignment with the CLAS concept (16, 17) and the four-step approach (8).

#### Step 1: Defining the Domains to Document

In a first step, the CPT defined the domains (or ICF categories) based on the ICF Generic-7 and−30 Sets (11, 12), the results of the situation analysis (3, 27) and in line with the guidelines on outcome measures of the DMGP. (23, 24). The CPT and the EPT decided to select assessment tools that are able to measure the defined ICF categories. If no assessments were available to cover specific categories, the patient's status in that category would be narratively described.

#### Step 2: Deciding What Perspective to Take

The CPT and EPT prioritized clinical and health care professional (HCP)-administered assessment tools to measure the ICF category, as these were deemed objective measures of functioning. To reflect the patient's perspective, patient-reported outcome measures (PROMs) were also selected. Associated with perspective is the decision about which profession(s) are responsible for assessing which aspect of functioning. In turn, this also guided the decision on the assessment tools to include. In some cases, more than one profession was defined as responsible.

#### Step 3: Identifying What Data Collection Tools to Apply

Identifying the data collection tools to apply mirrors the project's aim, i.e., to develop the Nottwil Standard. In addition to applying the results of steps 1 and 2, this step considered the results of the situational analysis, specifically which assessment tools were employed to measure different aspects of patient functioning and health, how and how often assessment tools were used, as well as the recommended toolkit defined in the DMGP guidelines (24) and the assessment tools recommended in the SwiSCI and EMSCI studies. Furthermore, suggestions for additional assessment tools recommended by HCPs based on recent scientific evidence were also considered.

#### Step 4: Deciding on When to Assess

The CPT defined the timepoint for assessment with the CLAS recommendation of the UEMS-PRM (16), the recommended timepoints from the SwiSCI (4, 12, 24 weeks post-injury and discharge) (29) and the EMSCI (1, 4, 12, 24, 48 weeks post-injury) (30) studies in mind.

### Toward Implementation of the Nottwil Standard

The implementation of the Nottwil Standard was documented in an implementation plan that outlined its integration in routine practice and in the existing documentation system, and the meetings with the EPT and all involved professional groups (physicians, therapists and nurses). To facilitate the integration of the Nottwil Standard in the clinic's electronic documentation system, documentation and process-based management representatives of the clinic's information technology department were also involved.

As Switzerland is a multi-language country, language diversity in the development of the Nottwil Standard was deemed important. Thus, where possible, PROMs in the Swiss languages of German, French, and Italian or English were included in the Nottwil Standard. These are the same languages used in the SwiSCI study (31).

To ensure the smooth implementation of the Nottwil Standard, a 1-year pilot study was conducted.

### Pilot Study

The pilot study was approved by the ethical committee (EKNZ Req-2020-01416) as a quality assurance project. The aim of the pilot implementation and quality assurance project was to evaluate the compliance with the standard. Data collection took place and included all patients admitted for initial rehabilitation after 1 July 2019 and discharged before 31 December 2020. Baseline patient characteristics, e.g., gender, age, completeness and level of lesion, as well as admission data, e.g., date of SCI onset and time of assessment, were recorded by the scientific assistant.

During the pilot study, the CPT regularly collected feedback and suggestions for improvement from the clinical staff beyond the EPT. The CPT and EPT discussed the collected input in two half-year feedback meetings, and the Nottwil Standard was adapted accordingly. The CPT and EPT regularly informed their respective clinical teams about changes that impacted the application of the Nottwil Standard during the pilot study.

## Results

### Situation Analysis

In total, 41 assessment tools were administered, 10 of which were administered more than once per patient. Of these 10 tools, the most frequently used were Spinal Cord Independence Measure III (SCIM) (32, 33), skin assessment and the Manual Muscle Test (34). The results show that outcome measures for motor activity, mobility and self-care were administered regularly, while measures for the autonomous nervous system, mental health and participation were not. Furthermore, neurological assessments like the International Standards for Neurological Classifications of SCI (ISNCSCI) (35) were not administered consequently nor at the recommended time points. Furthermore, the battery of assessment tools at the time did not cover the spectrum of categories of the ICF Core Set for post-acute care (3, 27).

### Defining the Domains to Document

The included ICF categories are presented in Table 1.

**Table 1:** Overview of the categories of the ICF Generic-30 Set (12). Post-acute SCI Set (13), and categories resulting from linking the Nottwil Standard to the ICF.

	**ICF Code and Label (G) = Category of the ICF Generic-7 Set (11)**	**ICF generic-30 Set**	**SCI Post-acute Brief**	**SCI post-acute comprehensive**	**Nottwil standard**
***N*** **=**		**30**	**27**	**52**	**61**
b114	Orientation functions				1
b126	Temperament and personality functions				1
b130	Energy and drive functions (G)	1		1	1
b134	Sleep functions	1		1	1
b137	Muscle power functions				1
b140	Attention functions				1
b144	Memory functions				1
b147	Psychomotor functions				1
b152	Emotional functions (G)	1	1	1	1
b156	Perceptual functions				1
b160	Thought functions				1
b164	Higher-level cognitive functions				1
b176	Mental function of sequencing				1
b180	Experience of self and time functions				1
b260	Proprioceptive function				1
b280	Sensation of pain (G)	1	1	1	1
b410	Heart functions				1
b415	Blood vessel functions				1
b420	Blood pressure functions				1
b430	Hematological system functions				1
b440	Respiration functions		1	1	1
b455	Exercise tolerance functions	1		1	1
b510	Ingestion functions				1
b525	Defecation functions		1	1	1
b530	Weight maintenance functions				1
b535	Sensations associated with the digestive system				1
b550	Thermoregulatory functions				1
b620	Urination functions	1	1	1	1
b640	Sexual functions	1		1	1
b665	Touch function				1
b710	Mobility of joint functions	1		1	1
b730	Muscle power functions	1	1	1	1
b735	Muscle tone functions		1	1	1
b770	Gait pattern functions				1
b810	Protective functions of the skin		1	1	1
d230	Carrying out daily routine (G)	1	1	1	
d240	Handling stress and other psychological demands	1	1	1	
d410	Changing basic body position	1	1	1	1
d415	Maintaining a body position	1	1	1	1
d420	Transferring oneself	1		1	1
d440	Fine hand use				1
d445	Hand and arm use		1	1	1
d450	Walking (G)	1	1	1	1
d455	Moving around (G)	1		1	1
d460	Moving around in different locations				1
d465	Moving around using equipment	1		1	1
d470	Using transportation	1		1	1
d475	Driving				1
d480	Riding animals for transportation				1
d510	Washing oneself	1	1	1	1
d520	Caring for body parts	1		1	1
d530	Toileting	1	1	1	1
d540	Dressing	1	1	1	1
d550	Eating	1	1	1	1
d560	Drinking		1	1	
d570	Looking after one's health	1		1	
d640	Doing housework	1		1	
d660	Assisting others	1		1	
d710	Basic interpersonal interactions	1		1	
d770	Intimate relationships	1		1	
d850	Remunerative employment (G)	1		1	1
d920	Recreation and leisure	1		1	1
e110	Products or substances for personal consumption			1	
e115	Products and technology for personal use in daily living		1	1	1
e120	Products and technology for personal indoor and outdoor mobility and transportation		1	1	1
e135	Products and technology for employment			1	
e150	Design, construction and building products and technology of buildings for public use			1	
e155	Design, construction and building products and technology of buildings for private use			1	
e225	Climate			1	
e310	Immediate family		1	1	
e320	Friends			1	
e340	Personal care providers and personal assistants		1	1	
e355	Health professionals		1	1	
e450	Individual attitudes of health professionals			1	
e580	Health services, systems and policies			1	1
e650	Financial assets				1
s110	Structure of brain				1
s120	Spinal cord and related structures		1	1	1
s430	Structure of respiratory system		1	1	1
s610	Structure of urinary system		1	1	1
s710	Structure of head and neck region				1
s720	Structure of shoulder region				1
s730	Structure of upper extremity				1
	TOTAL	30	27	52	63

The Nottwil Standard does not contain assessment tools that cover the following categories of the ICF Generic-30 Set and ICF Core Set for SCI in post-acute care due to the lack of adequate and established assessment tools that measure these categories: d230 Carrying out daily routine, d240 Handling stress and other psychological demands, d570 Looking after one's health, d640 Doing housework, d660 Assisting others, d710 Basic interpersonal interactions and d770 Intimate relationships.

### Deciding What Perspective to Take

The following professions were defined as those responsible for administering the Nottwil Standard: physicians (paraplegiology, neurology, urology, radiology, gynecology, pain management, hand surgery), nursing, physiotherapy, occupational therapy, psychology, social service, speech therapy, nutritional therapy, vocational counselor. See Table 2 for the list of assessment tools and responsible professions. Two PROMs, the Hospital Anxiety and Depression Scale (HADS) (36) and the SCI Quality of Life Basic Data Set (37), were also included in the Nottwil Standard.

**Table 2:** The Nottwil Standard assessment tools, responsible professions and compliance of all assessments during the pilot study.

**ICF Title**	**Diagnosis**	**Profession**	**Administered admission total (%)**	**Administered ITP total (%)**	**Administered discharge total (%)**	**Total adherence (%)**
NC	Etiology	Physician (Paraplegiology)	96 (100)			100
s120 Spinal cord and related structures	Lesion level	Physician (Paraplegiology)	96 (100)			100
**ICF Title**	**Structure/Function**	**Profession**	**Administered admission total (%)**	**Administered ITP Total (%)**	**Administered discharge total (%)**	**Total adherence (%)**
b665 Touch function b280 Sensation of pain b137 Muscle power function	ISNCSCI	Physician (Paraplegiology/Neurology)	96 (100)		94 (98)	>95
b410 Heart functions b415 Blood vessel functions b420 Blood pressure functions b620 Urination functions b525 Defecation functions b550 Thermoregulatory functions b640 Sexual functions	ISAFSCI	Physician (Paraplegiology/Neurology/Urology)		22 (23)	33 (34)	>25
b420 Blood pressure functions	Tilt table test (over Th10)	Physician (Paraplegiology/Neurology)		27 (39)		>35
b525 Defecation functions b535 Sensations associated with the digestive system d530 Toileting	Defecation method (SCI Bowel Function Basic Data Set)	Physician (Paraplegiology/Urology)Nurse				
b610 Urinary excretory functions b620 Urination functions b630 Sensations associated with urinary functions d530 Toileting	Bladder emptying (SCI Lower Urinary Tract Function Basic Data Set)	Physician (Paraplegiology/Urology)Nurse				
b620 Urination functions	Urodynamics/Uroflowmetry	Physician (Urology)		81 (84)95 (99)		>90
s610 Structure of urinary system	Ultrasound Bladder/Kidney	Physician (Urology)		95 (99)		>95
s120 Spinal cord and related structures	Electrophysiology	Physician (Neurology)		95 (99)		>95
b710 Mobility of joint functions	Range of Motion Lower Extremities	Physiotherapist	95 (99)		90 (94)	>95
b710 Mobility of joint functions	Range of Motion Upper Extremities	Physiotherapist	88 (92)		86 (90)	>90
b730 Muscle power functions	Manual Muscle Test Lower Extremities	Physiotherapist	95 (99)		87 (91)	>90
b280 Sensation of pain s720 Structure of shoulder region d420 Transferring oneself d460 Moving around in different locations d465 Moving around using equipment	WUSPI	Physiotherapist			54 (56)	>50
b114 Orientation functions b140 Attention functions b144 Memory function sb156 Perceptual functions b160 Thought functions b164 Higher-level cognitive functions b176 Mental function of sequencing complex movements	MoCA	Physician (Paraplegiology) Neuropsychology		71 (74)		>70
b410 Heart functions	ECG	Physician (Paraplegiology)	93 (97)			>95
b420 Blood pressure functions	Blood Pressure	Physician (Paraplegiology)	96 (100)		96 (100)	100
b440 Respiration functions	Lung Function: Spirometry/Bodyplethismography	Physician (Paraplegiology)		74 (77)18 (19)		>40
s120 Spinal cord and related structures	MRI (whole spine)	Physician (Paraplegiology/Radiology)		87 (91)		>90
s710 Structure of head and neck region	MRI (head)	Physician (Paraplegiology/Radiology)		71 (74)		>70
NC	Height	Nurse	79 (82)			>80
b530 Weight maintenance functions	Body Weight	Nurse	86 (90)		86 (90)	>90
NC	Leg Circumference	Nurse	96 (100)		95 (99)	>95
b810 Protective functions of the skin	Pressure Injury (yes/no)	Physician (Paraplegiology)				
b280 Sensation of pain	NRS Pain	Physician (Paraplegiology)		^*^17times applied	91 (94.79)	
b280 Sensation of pain	ISCIP	Physician (Paraplegiology)		11 (64.71)	95 (98.96)	
b280 Sensation of pain	SCIPI	Physician (Paraplegiology)		11 (64.71)	95 (98.96)	
b735 Muscle tone functions	Modified Ashworth Scale	Physiotherapist		30 (31.25)	57 (59)	
s770 Additional musculoskeletal structures related to movement	Osteoporosis/Densitometry und Bodycomposition	Physician (Paraplegiology/Radiology)		54 (56)		>50
NC	Vitamin D Status	Physician (Paraplegiology)		95 (99)		>95
b530 Weight maintenance functions	SNST	Nutrition Therapy	79 (82)			>80
See below	SCIM III	Nurse/Physiotherapist/ Occupational Therapist	96 (100)		96 (100)	100
d510 Washing oneself d520 Caring for body parts d540 Dressing d550 Eating	Self-Care	Nurse	96 (100)		96 (100)	
b620 Urination functions b525 Defecation functions d530 Toileting	Respiration and sphincter management	Nurse	96 (100)		96 (100)	
d420 Transferring oneself d450 Walking d455 Moving around d460 Moving around in different locations	Mobility (Room and Toilet)	Occupational therapist	96 (100)		96 (100)	
d420 Transferring oneself d450 Walking d455 Moving around d460 Moving around in different locations	Mobility (Indoors and Outdoors, on even Surface)	Physiotherapist	96 (100)		96 (100)	
b770 Gait pattern functions d450 Walking	WISCI II	Physiotherapist			59 (61)	>60
d465 Moving around using equipment	AMR (if in wheelchair)	Physiotherapist			47 (48.96)	>50
b455 Exercise tolerance functions	Endurance test	Sports medicine		41 (43)25 (26)		>30
e115 Products and technology for personal use in daily living e120 Products and technology for personal indoor and outdoor mobility and transportation d850 Remunerative employment	ANQ Goals	Physician (Paraplegiology)	96 (100)			100
**ICF Title**	**Quality of life**	**Profession**	**Administered admission total (%)**	**Administered ITP total (%**	**Administered discharge total (%)**	**Total adherence (%)**
b130 Energy and drive functions b126 Temperament and personality functions b152 Emotional functions b147 Psychomotor functions b160 Thought functions b180 Experience of self and time functions	HADS	Psychology			40 (42)	>40

*NC, Not covered by the ICF; ISNCSCI, International Standards for Neurological Classification of SCI; ISAFSCI, International Standard of Autonomic Function in Spinal Cord Injury; WUSPI, Wheelchair User's Shoulder Pain Index; MoCA, Montreal Cognitive Assessment; ECG, Electrocardiogram; MRI, Magnetic Resonance Imaging; NRS, Numeric Rating Scale; ISCIP, International Spinal Cord Injury Pain classification; SCIPI, Spinal Cord Injury Pain Instrument; SNST, Spinal Nutrition Screening Tool; SCIM, Spinal Cord Independence Measure; WISCI, Walking Index for Spinal Cord Injury; AMR, Aktivitätstest zur Mobilität im Rollstuhl (Activity test for Mobility in Wheelchair); ANQ, Swiss National Association for Quality Development in Hospitals and Clinics; HADS, Hospital Anxiety and Depression Scale; WHOQoL-BREF, World Health Organization Quality of Life*.

* *Real number without complete screening of pain incidence*.

### Identifying What Data Collection Tools to Apply

The Nottwil Standard version used for the pilot study contained 10 clinical examinations, 23 assessment instruments and two questionnaires. In addition, 7 tools were added specifically for the assessment of patients with tetraplegia (Table 3) and 5 tools to assess patients with walking ability (Table 4). Several assessment tools recommended in the DGMP guidelines and by the HCPs were also included: the International Standard of Autonomic Function in Spinal Cord Injury (ISAFSCI) (38), Spinal Cord Injury Pain Instrument (SCIPI) (39), International Spinal Cord Injury Pain classification (ISCIP) (40), 10 Meter Walk Test for Spinal Cord Injury (10 MWT) (41, 42) and WHO-QoL BREF (43), the Aktivitätstest zur Mobilität im Rollstuhl (activity test for mobility in wheelchair; AMR) (44, 45), the Bogenhausener Dysphagia Score (BODS) (46), and magnetic resonance imaging (MRI) of the brain and spinal cord. The Nottwil Standard tools were organized according to the ICF components of body functions and structures and activities and participation and ICF categories, and quality of life. See Tables 2–4.

**Table 3:** The Nottwil Standard assessment tools, responsible professions and compliance of all assessments for 42 patients with tetraplegia during pilot study.

**ICF Title**	**Structure/Function**	**Profession**	**Administered admission total (%)**	**Administered ITP total (%)**	**Administered discharge total (%)**	**Total adherence (%)**
b730 Muscle power functions	Manual muscle test upper extremities	Occupational therapist	42 (100)		41 (98)	>95
b730 Muscle power functions s730 Structure of upper extremity d445 Hand and arm use	ICSHT	Hand surgery		36 (89)		>85
b710 Mobility of joint functions s730 Structure of upper extremity	Range of motion wrist-finger	Occupational therapist	41 (98)		33 (84)	>90
b730 Muscle power functions s730 Structure of upper extremity	Jamar hand dynamometer	Occupational therapist	23 (41.82)		19 (34.55)	
s730 Structure of upper extremity d440 Fine hand use d445 Hand and arm use	GRASSP 2	Occupational therapist	42 (100)		37 (91)	>95
b430 Hematological system functions	Pulse oximetry	Physician (Paraplegiology)	41 (98)			>95
b510 Ingestion functions	BODS	Physician (Paraplegiology) Logopedics	41 (98)		40 (96)	>95

*ICSHT, International Classification for Surgery of the Hand in Tetraplegia; GRASSP, Graded Redefined Assessment of Strength, Sensibility and Prehension; BODS, Bogenhausener Dysphagia Score*.

**Table 4:** The Nottwil Standard assessment tools, responsible professions and compliance of all assessments for patients with walking ability during the pilot study.

**ICF Title**	**Structure/Function**	**Profession**	**Administered admission total (%)**	**Administered ITP total (%)**	**Administered discharge total (%)**	**Total adherence (%)**
b260 Proprioceptive function	Deep proprioceptive sensitivity	Physician (Paraplegiology/Neurology)				
b770 Gait pattern functions d450 Walking	10 meter walk test	Physiotherapist			13 (42)	>40
b770 Gait pattern functions d450 Walking	Six-minute walk test	Physiotherapist			28 (90)	>90
d410 Changing basic body position d450 Walking	Timed up and go test	Physiotherapist			22 (71)	>70
b770 Gait pattern functions	Gait analysis	Physiotherapist/Neurology				

### Deciding on When to Assess

The time points for conducting the assessment were defined as follows: admission (0–2 weeks after admission) and discharge (0–3 weeks before discharge). For specific assessment tools, additional time points were considered clinically relevant. See Table 2.

### Toward Implementation of the Nottwil Standard

Other than the assessment tools recommended by the DMGP guidelines (i.e., ISAFSCI, SCIPI, ISCIP, 10 MWT, WHO-QoL BREF) or the HCPs (i.e., AMR, BODS and MRI), the assessment tools included in the Nottwil Standard had already been in routine use in the clinic and integrated in the HIS. The newly introduced assessments were initially introduced in paper form. The ISAFSCI was later integrated into the clinic's HIS.

In terms of the controlling mechanism during the pilot study, when an assessment was not conducted as described in the Nottwil Standard, reminders were manually sent to the responsible person(s), and reasons for non-performance were documented in the HIS for the specific patient. This documentation was visible to all clinical staff involved in the rehabilitation of that patient. The development of an automated reminder system is planned.

### Pilot Study

Forty-two patients with tetraplegia and 54 patients with paraplegia (75 were male and 21 female) were included in the pilot study.

All senior (*n* = 10) and junior (*n* = 19) physicians, 40 physiotherapists, 20 occupational therapists, 8 psychologists, 5 nutrition specialists, 6 social workers participated in the training on the Nottwil Standard. While many of the assessment tools were already in routine use, physicians had to learn to use the ISAFSCI, ISCIP and SCIPI, and physiotherapists had to be trained to use the 10 MWT.

The following assessments were administered at a 100% compliance level in accordance with the Nottwil Standard recommendations: ISNCSCI, urological examination, manual muscle tests, range of motion testing and SCIM III. See Table 2. The lowest compliance rates were observed for ISAFSCI (35%), ISCIP (35%), SCIPI (35%), (HADS 41%). Assessments using WHOQOL BREF was not implemented at all.

In summary, the Nottwil Standard (see Tables 2–4) covers the complexity of functioning associated with SCI, including but not limited to neuromuscular functions (e.g., 10 MWT, manual muscle test), functions of the autonomic nervous system (e.g., ISAFSCI, blood pressure), pain (e.g., numeric rating scale for pain), mental/psychological functions (e.g., HADS), bladder and bowel management (e.g., SCIM III), mobility (e.g., AMR), participation in work and social life (e.g., SCIM III, ANQ goals), influence of assistive devices (e.g., ANQ goals). Since the functioning of persons with SCI can differ greatly depending on whether the person is living with paraplegia or tetraplegia, or can or cannot walk, the Nottwil Standard also contains specific assessment tools for these sub-populations of persons with SCI.

## Discussion

In this paper, we reported on the development of the Nottwil Standard, an ICF-based assessment standard for use in the rehabilitation of persons after newly acquired SCI and on the first experiences in implementing it. Developing the Nottwil Standard not only met the challenge of determining a battery of tools that support a comprehensive and accurate assessment of health and functioning of patients with SCI, it showed that it is feasible to develop and implement it in an interprofessional and participatory manner. Furthermore, this project reflects the potential for real-life clinical application of the ICF that also promotes the clinical quality management.

### Potential for Real-Life Clinical Application of the ICF

Although the ICF was launched in 2001, the implementation of the ICF in the clinical management of individuals with SCI took over a decade. This is consistent with findings of a mixed method examination of the extent of ICF diffusion in clinical rehabilitation (not only SCI) between 2001 and 2010. This study showed that clinical implementation of the ICF at the time was rare and called for more large-scale research to address the need for best practice recommendations implementing the ICF in clinical rehabilitation (47). In terms of SCI care, there were early implementation efforts, e.g., in developing an ICF-based electronic tool for use in the long-term clinical follow-up of patients with SCI (48). The ICF has also been key in framing outcomes in SCI care, including the International SCI data sets (49). Efforts to implement the ICF in SCI rehabilitation gained momentum with the development of the ICF Core Set for SCI for the post-acute care and the ICF Generic-30 Set (12, 13), For example, the DGMP guidelines for outcome measures in inital rehabilitation after the onset of a SCI calls for using the ICF Core Set for SCI in post-acute care in selecting the outcome measures to use (24). This was one of the drivers for developing the Nottwil Standard.

The ICF and its underlying comprehensive biopsychosocial perspective also stimulated the decision to include the additional assessment tools suggested by the HCP, i.e., ISAFSCI for autonomic functioning, SCIPI and ISCIP for pain examination, AMR for wheelchair mobility, BODS for swallowing function, the brain and spinal MRI for nerve structural changes, the Montreal Cognitive Assessment for neurocognitive functioning (50) and WHO-QoL for the quality of life evaluation. This helped to ensure that the Nottwil Standard comprehensively covered as many SCI-relevant functioning areas as possible. Ultimately, the aim of establishing such a comprehensive standard for assessing functioning and health of patients with SCI is to improve quality of care.

### Quality Management in SCI Rehabilitation

The value of employing the ICF in clinical quality management has been recognized at the national and international level. At the international level, the UEMS-PRM has developed the European Framework for rehabilitation service types and corresponding CLAS as ICF-based standards for improving rehabilitation quality in Europe (14–16). At the national level, the ANQ, the national organization responsible for ensuring quality hospitals and clinics in Switzerland, calls for using the ICF in participation goal-setting (20). For this reason, the Nottwil Standard includes participation goal-setting based on ANQ criteria. The ANQ also calls for employing specific functioning-based instruments for reporting outcomes to the ANQ for further developing and improving quality in Swiss hospitals and clinics. These instruments reflect concrete ICF categories (51).

Ensuring clinical quality in SCI rehabilitation goes beyond ICF implementation. The pilot study showed that the successful implementation of the Nottwil Standard requires the commitment at the institutional level, active involvement of clinicians and an effective information-sharing strategy. Active involvement of the clinicians encompassed training on the Nottwil Standard and continuous discussion, evaluation and adaptation of its use (ongoing). These discussions, the controlling mechanism put in place during the pilot study and the dissemination of information on the status of the pilot study and planned adaptations of the Nottwil Standard were all elements of the information-sharing strategy.

### Culture of Change

The implementation of the Nottwil Standard constitutes a change in the way assessments are done in the rehabilitation of newly injury patients with SCI. Managing change as a result of the Nottwil Standard can be viewed from the perspective of Kotter's eight steps of change management in health care: “increase urgency, building guiding teams, get the vision right, communicate for buy-in, enable action, create short-term wins, don't let up, make it stick” (52). The sense of urgency to develop and implement the Nottwil Standard has its roots in the DGMP guidelines (24) for outcome measures and ANQ goal-setting and outcomes reporting criteria (20), which promotes ICF implementation in rehabilitation, and has been building up with the results of the situation analysis (3, 27). Building guiding teams was satisfied with the establishment of a cohesive coalition between the CPT and the EPT and involving all relevant professions. Getting the vision right and communicating for buy-in are related. Clearly communicating the reasons for the Nottwil Standard and regularly communicating the status of the implementation plan were deemed as important and realized through regular meetings and information-sharing with clinical staff. Regular information-sharing and the active involvement in implementing the Nottwil Standard was the opportunity for clinical staff to connect the results of the assessments and the impact on clinical management. The HCP also had to learn that the Nottwil Standard does not hinder individualized management but rather promotes a comprehensive assessment of the patient while simplifying the complexity of the patient's health and functioning. Enabling action was reflected in the support of the clinic's management by investing necessary resources (setting up the CPT and EPT, time for meetings, engaging the IT department) in the project. Creating short-term wins can be seen in the acknowledgment of clinical staff that routine data-based assessments according to the Nottwil Standard was possible. The last two steps (don't let up, making change stick) are ongoing. The Nottwil Standard must undergo continuous improvement based on scientific developments and an evolving clinical and organization environment (7, 53, 54).

## Limitations

Some limitations are noteworthy to mention. First, despite efforts to achieve comprehensiveness of the Nottwil Standard by including assessment tools that cover as many SCI-relevant ICF categories as possible, no adequate assessment tools were found for seven categories of the combined ICF Generic-30 Set and ICF Core Set for SCI in post-acute care. Alternatives to established assessment tools for assessing these categories, e.g., single item-questions, will be explored. Second, the Nottwil Standard was developed for implementation in a specific rehabilitation facility. Applying the Nottwil Standard to other hospitals and clinics will require additional testing and possible adaptation. Nevertheless, the principles of continuous improvement can be used in different settings. Lastly, the controlling mechanism nor all the assessment tools had been integrated in the HIS during the pilot study. Thus, the results of the pilot study may have been influenced by this lack of an automated reminder system, as well as the inability to electronically extract relevant data from performed assessments. Respective updates to the HIS are ongoing.

## Conclusion

The Nottwil Standard is an ICF-based assessment standard for a comprehensive and accurate assessment of health and functioning of persons after newly acquired SCI. It was developed by an interprofessional group of rehabilitation professionals in a consensus-oriented collaborative process and guided by a set of principles. The Nottwil Standard can be feasibly integrated in routine practice and in the existing HIS. Implementation also requires a well-structured process with a good communication strategy and controlling mechanism, and full engagement of the involved multiprofessional clinical staff. Further development activities include the integration of all the Nottwil Standard assessment tools in the clinic's HIS and deciding on how to assess the SCI-relevant ICF categories that the Nottwil Standard should cover but for which no assessment tool had yet been found. Lastly, since the ultimate aim of the Nottwil Standard is the continuous improvement of rehabilitation quality, an evaluation of impact of the Nottwil Standard on care quality, for example in terms of patient and staff satisfaction, is warranted.

## Data Availability Statement

The original contributions presented in the study are included in the article/supplementary material, further inquiries can be directed to the corresponding author.

## Ethics Statement

The ethical committee Ethikkommission Nordwestschweiz (EKNZ Req-2020-01416) approved the study. The patients/participants provided their written informed consent to participate in this study.

## Author Contributions

PL and AS-S collected the data and were responsible for the methodological quality. MS, PL, and AS-S drafted the first version of the manuscript and all authors approved the last version and gave feedback to the final manuscript. All authors drafted the study, the development and implementation of the Nottwil standard.

## Conflict of Interest

The authors declare that the research was conducted in the absence of any commercial or financial relationships that could be construed as a potential conflict of interest. The handling editor declared a past collaboration with several of the authors MS and GS.

## Publisher's Note

All claims expressed in this article are solely those of the authors and do not necessarily represent those of their affiliated organizations, or those of the publisher, the editors and the reviewers. Any product that may be evaluated in this article, or claim that may be made by its manufacturer, is not guaranteed or endorsed by the publisher.

## Abbreviations

SCI: Spinal cord injury.
